# Comparative analysis of the metabolites and biotransformation pathways of fentanyl in the liver and brain of zebrafish

**DOI:** 10.3389/fphar.2023.1325932

**Published:** 2023-12-18

**Authors:** Meng Liu, Jian Huang, Sen Zhao, Bin-jie Wang, Hong Zhou, Yao Liu

**Affiliations:** ^1^ School of Investigation, People’s Public Security University of China, Beijing, China; ^2^ School of Investigation, Zhejiang Police College, Hangzhou, China; ^3^ Key Laboratory of Drug Prevention and Control Technology of Zhejiang Province, Zhejiang Police College, Hangzhou, China; ^4^ Institute of Forensic Science, Ministry of Public Security, Beijing, China

**Keywords:** fentanyl, metabolites, biotransformation pathways, the liver and brain of zebrafish, HR-MS

## Abstract

The rise of fentanyl has introduced significant new challenges to public health. To improve the examination and identification of biological samples in cases of fentanyl misuse and fatalities, this study utilized a zebrafish animal model to conduct a comparative investigation of the metabolites and biotransformation pathways of fentanyl in the zebrafish’s liver and brain. A total of 17 fentanyl metabolites were identified in the positive ion mode using ultra-high-pressure liquid chromatography Q Exactive HF Hybrid Quadrupole-Orbitrap mass spectrometry (UHPLC-QE HF MS). Specifically, the zebrafish’s liver revealed 16 fentanyl metabolites, including 6 phase I metabolites and 10 phase II metabolites. Conversely, the zebrafish’s brain presented fewer metabolites, with only 8 detected, comprising 6 phase I metabolites and 2 phase II metabolites. Notably, M′4, a metabolite of dihydroxylation, was found exclusively in the brain, not in the liver. Through our research, we have identified two specific metabolites, M9-a (monohydroxylation followed by glucuronidation) and M3-c (monohydroxylation, precursor of M9-a), as potential markers of fentanyl toxicity within the liver. Furthermore, we propose that the metabolites M1 (normetabolite) and M3-b (monohydroxylation) may serve as indicators of fentanyl metabolism within the brain. These findings suggest potential strategies for extending the detection window and enhancing the efficiency of fentanyl detection, and provide valuable insights that can be referenced in metabolic studies of other new psychoactive substances.

## 1 Introduction

The emergence of fentanyl analogs has significantly impacted the field of pharmacology and presented new challenges in the realm of public health (Clotz et al., 1991; Poklis, 1995; Zawilska et al., 2017; Han et al., 2019). Fentanyl is a synthetic opioid that is approximately 50–100 times more potent than morphine, its high potency, fast onset of action, and duration of the desired effect may be particularly important contributing factors to the higher risk of overdose deaths and social consequences. Additionally, it is frequently combined with other substances like heroin or cocaine, often unwittingly to the user. This scenario makes it exceedingly challenging for users to accurately assess the strength of the substances they are consuming, consequently leading to a rise in fentanyl-related overdose deaths (Henderson, 1991; Hibbs et al., 1991; Nolan et al., 2019). Survey data from New York City, West Virginia, and Australia indicate that since 2015, there has been a significant rise in the rate of fentanyl-related overdose deaths worldwide. Illicitly manufactured fentanyl and its analogues have become some of the primary substances identified in these fatalities (Colon-Berezin et al., 2019; Dai et al., 2022; Roxburgh et al., 2022).

Delving into the metabolites and biotransformation pathways of fentanyl provides a broader perspective on its toxicological impacts. Pinpointing its signature metabolites broadens the detection time frame, consequently enhancing the efficiency of tests. This is key in keeping a vigilant eye on potential fentanyl abuse. In recent years, the focus of research has increasingly centered on the qualitative and quantitative analysis of trace amounts of fentanyl and its metabolites in biological samples (Strayer et al., 2018; Busardò et al., 2019; Ott et al., 2022; Wei et al., 2022). The majority of reported fentanyl metabolites are phase I metabolites, which include N-dealkylation and hydroxylation metabolites. While phase II metabolites, which are primarily glucuronidation metabolites, are not as commonly reported. Consequently, there is a need for more research to broaden our understanding of phase II metabolism.

Zebrafish models offer several advantages over experimental rodent models, including greater time efficiency, cost-effectiveness, high-throughput capacity and sensitivity. Adhering to the internationally recognized 3Rs (replacement, reduction, refinement) principle in toxicological experiments, zebrafish models also boast a genome strikingly similar to that of humans. Furthermore, zebrafish possess a metabolic enzyme system that closely resembles that of mammals. Enzymes from the CYP450 family play a crucial role in the initial phase of drug metabolism in mammals. Goldstone et al. demonstrated that many zebrafish CYP450 enzymes have direct orthologues in humans and other mammals, underscoring the relevance of zebrafish as a model for studying drug metabolism. Notably, there are unique CYPs in fish, such as CYP1C, CYP2AE, and CYP2X, which lack human orthologs, suggesting potential differences in metabolic pathways (Goldstone et al., 2010). Furthermore, the gene expression profiles of phase II enzymes, including uridine glucuronosyltransferases (UGTs), sulfotransferases (SULTs), and methyltransferases (COMTs) in zebrafish, have been characterized and show similarities to their human counterparts (Alazizi et al., 2011; Mohammed et al., 2012; Christen et al., 2014). Consequently, zebrafish are capable of performing both phase I (oxidation, N-demethylation, O-demethylation, and N-dealkylation) and phase II (sulfation, glucuronidation, and methylation) drug metabolism reactions, paralleling human processes (Matos et al., 2020; Pesavento et al., 2022).

Zebrafish, frequently dubbed as “little mice in water” have garnered considerable interest in recent years. Their employment as biological models to investigate drug toxicity and metabolism has propelled them to the forefront of research (Xu et al., 2019; Morbiato et al., 2020; Krishna et al., 2021; Cooman et al., 2022b; Pesavento et al., 2022) conducted a study on the metabolism of fentanyl in zebrafish embryos, but no research has been reported on fentanyl metabolism in adult zebrafish. Fentanyl is a lipophilic compound, can swiftly cross the cell membrane and penetrate the blood-brain barrier to exert its medicinal effects within the brain (Yu et al., 2022). The liver, as the primary organ for drug metabolism, is abundant in drug-metabolizing enzymes.

For this study, the metabolites and biotransformation pathways of fentanyl in the liver and brain of zebrafish were examined and identified using ultra-high-pressure liquid chromatography Q Exactive HF Hybrid Quadrupole-Orbitrap mass spectrometry (UHPLC-QE HF MS), with an emphasis on the analysis of Phase II metabolites. This approach lays a solid foundation for the examination and identification of biological samples in cases of fentanyl misuse and fatalities.

## 2 Experimental

### 2.1 Drugs and reagents

Fentanyl hydrochloride standard with a purity of at least 99.5% was sourced from Shanghai Yuansi Standard Technology Co., Ltd. in Shanghai, China, and was dissolved in ultrapure water to create a 10 mgL^−1^ (26.8 μM) fentanyl solution for later use. Chromatographically pure acetonitrile and formic acid were purchased from Merck & Co., Inc. In New Jersey, United States.

### 2.2 Zebrafish study

All zebrafish experimental procedures were reviewed and approved by Zhejiang University Experimental Animal Welfare. In our experiment, we worked with adult wild-type zebrafish of the AB strain, which we sourced from the Wuhan Zebrafish Center in China. We kept them in a recirculating tank system (RTS), courtesy of Shanghai Haisheng Biotechnology. This system kept the water clean and well-oxygenated, with a pH of around 7.2, conductivity from 500 to 600 μS, and a cozy temperature between 27.5°C and 28.5°C. The zebrafish enjoyed a diet of freshly hatched brine shrimp served twice a day, and we mimicked their natural light/dark cycle with 14 h of light followed by 10 h of darkness. Before we introduced them to the bath treatment for our metabolic studies, we let them get used to the tank system for at least a week.

Eighteen adult zebrafish (4–6 months old, weighing 0.5 ± 0.1 g) were divided into two groups of nine. A couple of hours before the experiment kicked off, we transferred both groups from the RTS to Petri dishes, each containing 30 mL of RTS water. This was to help them acclimate. We put three fish in each dish. One group was chosen for drug exposure. When the experiment started, we replaced the water in their RTS with an equal volume of fentanyl aqueous solution (10 mgL^−1^, 26.8 μM), and the fish were exposed to this solution for 24 h. The other group served as a control, remaining in the RTS water without drug exposure for the same period.

After the bath administration period, the zebrafish were rinsed thrice with ultrapure water, then humanely euthanized in grinding tubes. The livers were then harvested, with every three livers from the same group pooled together. Next, three grinding beads and 200 µL of acetonitrile were added to each tube, followed by homogenizing the samples using a JXFSTPRP-CL fully automatic sample freezing and grinding machine (Shanghai JingXin Co., Ltd., China) at −4°C (a frequency of 60 Hz, a run time of 40 s, and a pause time of 20 s, for a total of 10 cycles). This was followed by centrifugation at 13,000 r/min and 4°C for 10 min using a Legend Micro 21R freezing high-speed centrifuge (Thermo Fisher Scientific, Waltham, MA, United States). The supernatant was collected, re-dissolved using a nitrogen blow, and then filtered through a 0.22 μm organic PTFE microporous membrane into a lined tube for subsequent instrument analysis.

The treatment was similarly administered to the zebrafish brain.

### 2.3 Instrumental analysis

All samples were analyzed using a by ultra-high-pressure liquid chromatography Q Exactive HF Hybrid Quadrupole-Orbitrap mass spectrometry (Thermo Fisher Scientific, Waltham, MA, United States). The chromatographic column was a ACQUITY UPLC HSS T3 Column (150 mm × 2.1 mm, 1.8 μm) fitted with a VanGuard precolumn, both from Waters (Milford, MA, United States). Mobile phases were 0.1% formic acid (A) and 0.1% formic acid in acetonitrile (B) and were run in a gradient at a flow rate of 0.3 mLmin^−1^ starting at 1% B until 1.0 min, ramped to 99% B at 8.0 min and held until 10.0 min, then ramped down to 1% B at 10.1 min and finally re-equilibration until 12 min. The column temperature was kept at a steady temperature of 30°C, and an injection volume of 3 µL was maintained.

An electrospray ionization source (ESI) was utilized that operated at a spray voltage of 3,800 V. Nitrogen was used as the collision gas, and the atomization temperature was set at 320°C, an atomization gas pressure of 38 arb was maintained and an auxiliary gas pressure of 15 arb, with a capillary temperature of 350°C. Data acquisition was performed using the Full MS-ddMS^2^ scan mode under the following conditions: a Full MS resolution of 35,000, a maximum injection time of 100 m, and a scan range of *m/z* 70–1,000. For tandem MS data collection, the dd-MS^2^ resolution was 17,500, with collision energies set at 17.5, 35, and 52.5 eV.

## 3 Results and discussion

### 3.1 Mass spectrometry analysis of fentanyl

**FIGURE 1:**
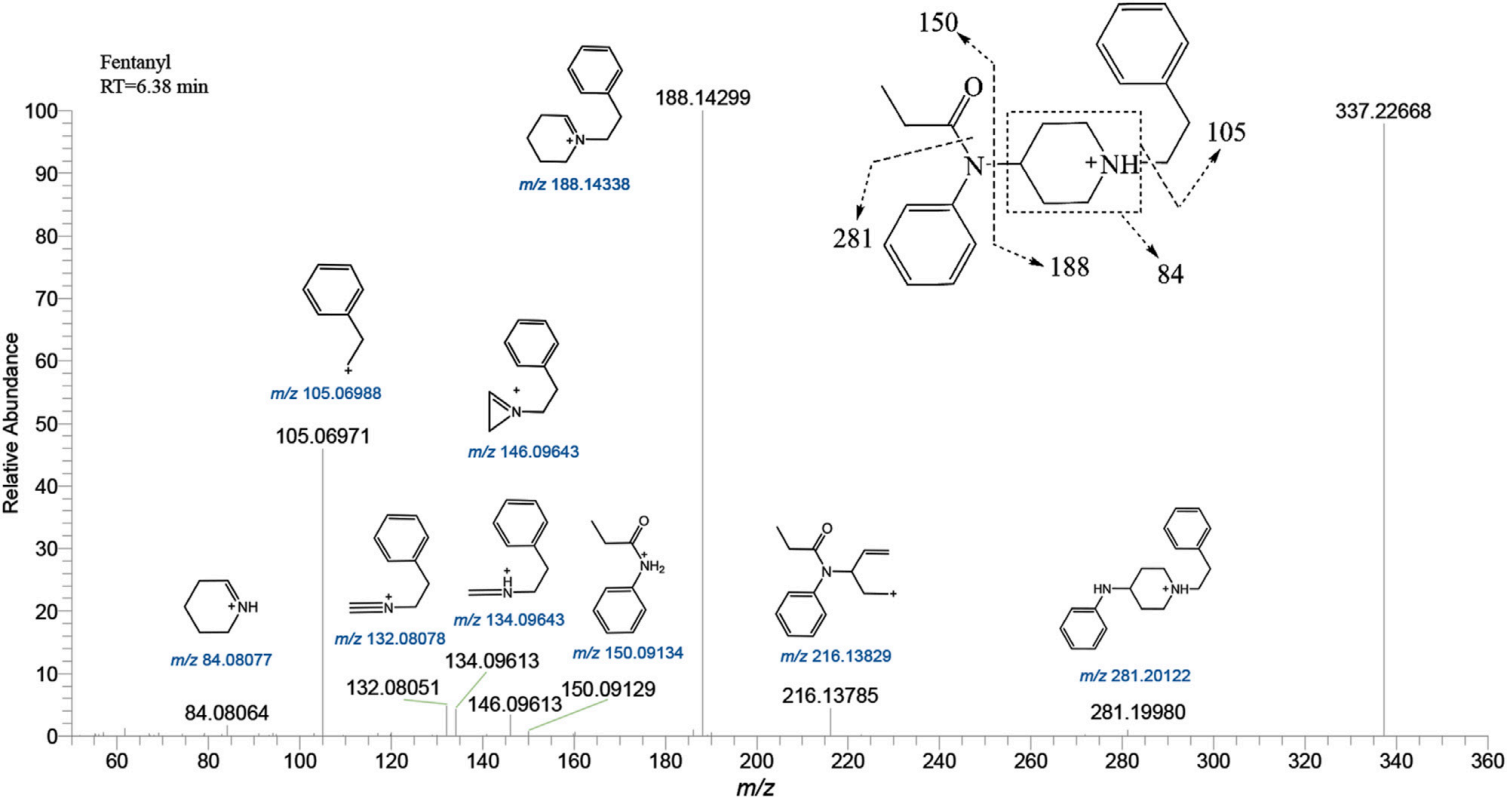
Mass spectra and assigned fragmentation patterns for fentanyl.

**FIGURE 2:**
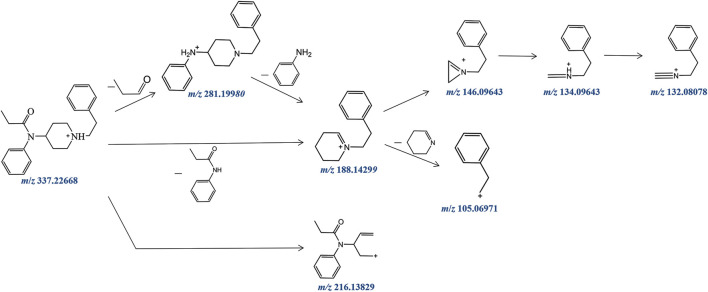
Fragmentation pathway of fentanyl in ESI-MS.

Fentanyl, with the molecular formula C_22_H_28_N_2_O, exhibited an accurate mass of the protonated molecular ion [M + H]^+^ at *m/z* 337.22744 under the positive ion collection mode. The MSMS spectrum of fentanyl with characteristic fragment structures is depicted in Figure 1. Referencing Davidson’s study (Davidson et al., 2022) on fentanyl’s mass spectrometry fragmentation patterns, we observed that the fragmentation of fentanyl’s mass spectrum adheres to the patterns of *m/z* 337→281→188→146→134→132, 337→188→105 and 337→216. The detailed MSMS spectrometry fragmentation pathways, which further elucidate these patterns, are illustrated in Figure 2.

### 3.2 Examination of fentanyl metabolites

**FIGURE 3:**
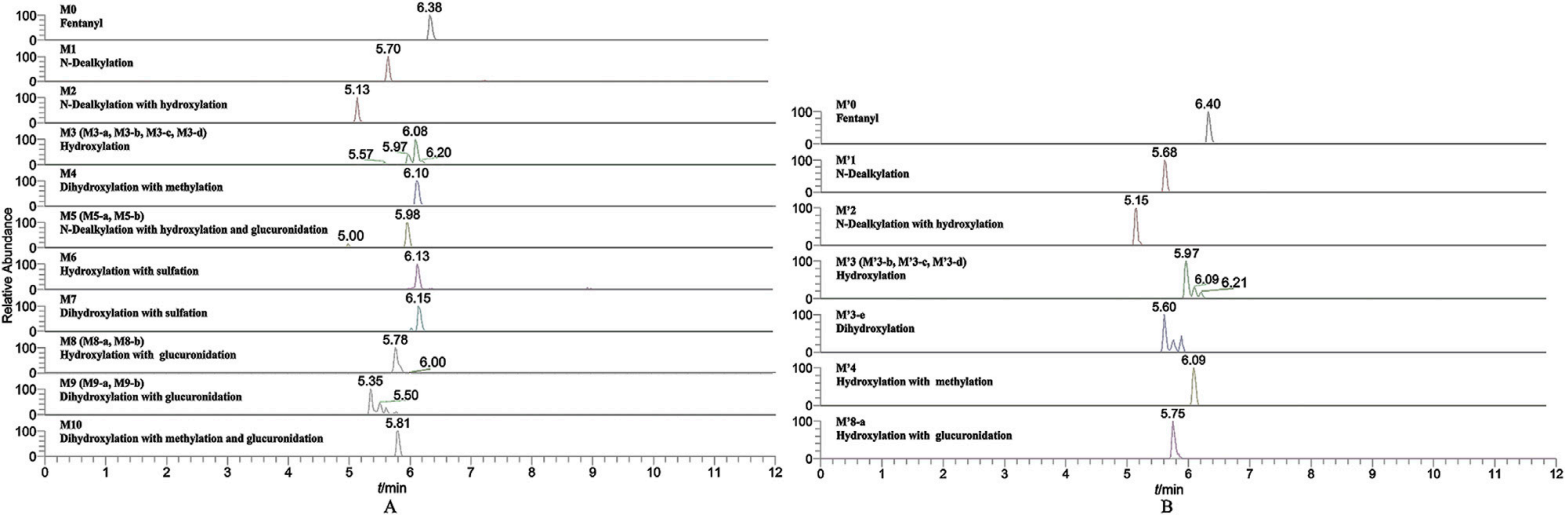
Extracted ion chromatogram for the major metabolites of fentanyl detected in the liver **(A)** and brain **(B)** of zebrafish.

In the positive ion mode, a total of 17 fentanyl metabolites were detected in the zebrafish’s liver and brain, M′4 was unique that it was only found in the brain but not in the liver. All other metabolites detected in the brain were identifiable in the liver of the zebrafish. To be specific, 16 fentanyl metabolites were detected in the zebrafish’s liver, comprising 6 phase I metabolites and 10 phase II metabolites. In contrast to the liver, the brain of zebrafish contained fewer metabolites, with a total of 8 detected. This included 6 phase I metabolites and 2 phase II metabolites, detailed in Table 1. Notably, glucuronidated fentanyl (M9-a) and monohydroxy fentanyl (M3-c) were the major metabolites in the liver. In the brain, however, norfentanyl (M1) and monohydroxy fentanyl (M3-b) were more prevalent. Phase I metabolites primarily consisted of dealkylation and hydroxylation products, while the phase II metabolites were predominantly the results of methylation, glucuronidation and sulfation processes. The extracted ion chromatogram of these metabolites is depicted in Figure 3, and the detailed information can be found in Table 2, mass error for protonated molecule of all these metabolites being less than 3.46 ppm. In comparison to earlier studies on fentanyl metabolism using hepatocytes (Kanamori et al., 2018a; Kanamori et al., 2018b) or zebrafish embryos (Pesavento et al., 2022), our study revealed a more diverse fentanyl metabolites, with a particular increase in phase II metabolites. Of significant note, this study presents the first report of fentanyl’s sulfation metabolites.

**TABLE 1:** Peak areas of fentanyl and metabolites detected in the liver and brain of zebrafish.

Metabolite	Liver (rank)	Brain (rank)	Metabolite	Liver (rank)	Brain (rank)
Fentanyl	2.14E9	3.33E9	M6-a	2.32E6 (15)	n.d
M1	2.08E8 (3)	3.21E8 (1)	M6-b	2.31E7 (7)	n.d
M2	1.16E7 (11)	7.07E6 (6)	M7	1.82E7 (8)	n.d
M3-a	1.57E7 (9)	n.d	M8	3.13E6 (14)	n.d
M3-b	9.28E7 (4)	1.35E8 (2)	M9-a	2.91E8 (1)	1.70E7 (5)
M3-c	2.19E8 (2)	4.05E7 (3)	M9-b	1.75E5 (16)	n.d
M3-d	2.78E7 (6)	2.11E7 (4)	M10-a	1.51E7 (10)	n.d
M’4	n.d	3.74E6 (7)	M10-b	8.39E6 (12)	n.d
M5	4.79E6 (13)	3.26E6 (8)	M11	6.21E7 (5)	n.d

The peak area rankings of metabolites given in the bracket; n. d. not detected.

**TABLE 2:** Detailed information of fentanyl and metabolites in the liver and brain of zebrafish.

Class	Biotransformation	RT/min	[M + H]^+^/(*m/z*)		Error/ppm	Characteristic fragment
			Calculated	Measured		
P	Parent drug	6.38	337.22744	337.22668	−2.25	188.14299, 105.06971, 216.13785, 132.08051, 134.09613, 146.09613
M1	N-Dealkylation	5.70	233.16484	233.16451	−1.41	84.08068, 150.09119, 177.13835
M2	N-Dealkylation with hydroxylation reaction	5.13	249.15975	249.15916	−2.37	84.08061, 166.08578, 177.13788, 94.06496, 73.02837
M3-a	Hydroxylation	5.57	353.22235	353.22174	−1.73	204.13811, 121.06465, 84.08067, 162.09114
M3-b		5.97		353.22131	−2.94	188.14288, 105.06965, 134.09612, 146.09596
M3-c		6.08		353.22156	−2.24	121.06454, 204.13794, 84.08063, 216.13773
M3-d		6.20		353.22137	−2.77	204.13783, 186.12727, 132.08047, 335.21075, 174.12726
M’4	Dihydroxylation	5.60	369.21727	369.21698	−0.785	121.06461, 204.13803, 84.08065, 232.13333
M5	Dihydroxylation with methylation reaction	6.10	383.23292	383.23230	−1.62	151.07515, 234.14851, 119.04902, 84.08069, 192.10179
M6-a	N-Dealkylation with hydroxylation and glucuronidation reaction	5.00	425.19184	425.19061	−2.89	249.15919, 84.08060, 166.08572, 177.13782
M6-b		5.98		425.19086	−2.30	82.06499, 249.15921, 233.16437, 150.09111, 100.07552
M7	Hydroxylation with sulfation reaction	6.13	433.17917	433.17767	−3.46	353.22153, 204.13794, 121.06456, 284.09439, 84.08062
M8	Dihydroxylation with sulfation reaction	6.15	449.17408	449.17343	−1.45	369.21661, 220.13290, 137.05951, 84.08064, 178.08575
M9-a	Hydroxylation with glucuronidation reaction	5.78	529.25444	529.25348	−1.81	121.06457, 353.22150, 204.13792, 84.08061, 380.16934
M9-b		6.00		529.25348	−1.81	353.22153, 204.13788, 186.12737, 335.21094, 134.09613
M10-a	Dihydroxylation with glucuronidation reaction	5.35	545.24936	545.24847	−1.63	121.06458, 204.13795, 369.21655, 380.16949, 84.08070
M10-b		5.50		545.24884	−0.954	204.13808, 369.21667, 121.06464, 186.12741, 351.20627
M11	Dihydroxylation with methylation and glucuronidation reaction	5.81	559.26501	559.26422	−1.41	151.07515, 383.23221, 234.14845, 192.10159, 84.08065

#### 3.2.1 Examination of phase I metabolites

Six phase I metabolites were identified in both the liver and brain of zebrafish, M3-a was exclusive to the liver, whereas M′4 was found only in the brain.

##### 3.2.1.1 Metabolites of N-Dealkylation

**FIGURE 4:**
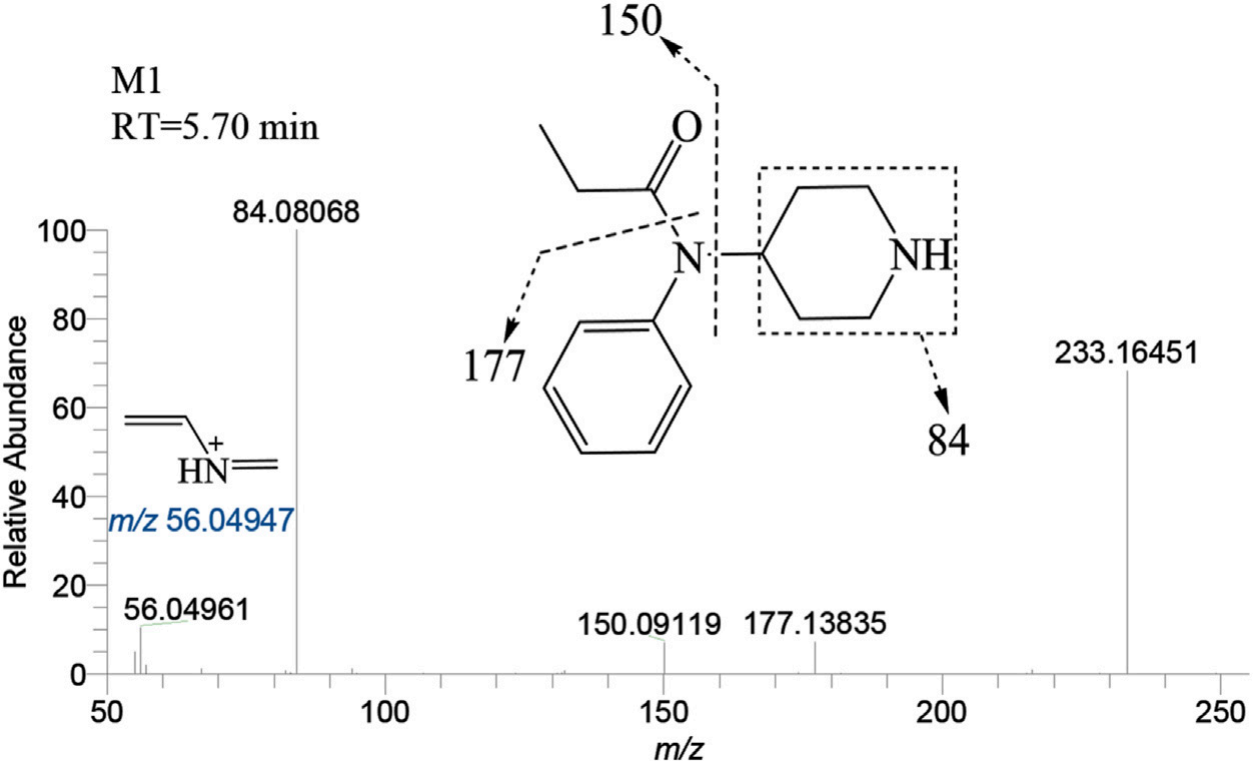
Mass spectra and assigned fragmentation patterns for M1.

The N-dealkylation metabolite on the piperidine ring, designated as M1and also referred to as norfentanyl, has been identified as a signature metabolite of fentanyl (Wilde et al., 2019; Armenian et al., 2022), was identified in both the liver and brain of zebrafish. With a retention time of 5.70 min, M1 formed an accurate mass of the protonated molecular ion [M + H]^+^ at *m/z* 233.16484 (C_14_H_20_N_2_O), showing a reduction of 104 Da (-C_8_H_8_) compared to the parent drug. The main characteristic fragment ions of M1 were *m/z* 84.08068, 150.09119, and 177.13835, as depicted in Figure 4. The characteristic fragments ions at *m/z* 84 and 150 align with the corresponding parent drug. However, the fragment at *m/z* 177 was 104 Da less than the parent drug at *m/z* 281, indicating that M1 was formed as a result of N-dealkylation on the piperidine ring of the parent drug.

##### 3.2.1.2 Metabolites of hydroxylation

**FIGURE 5:**
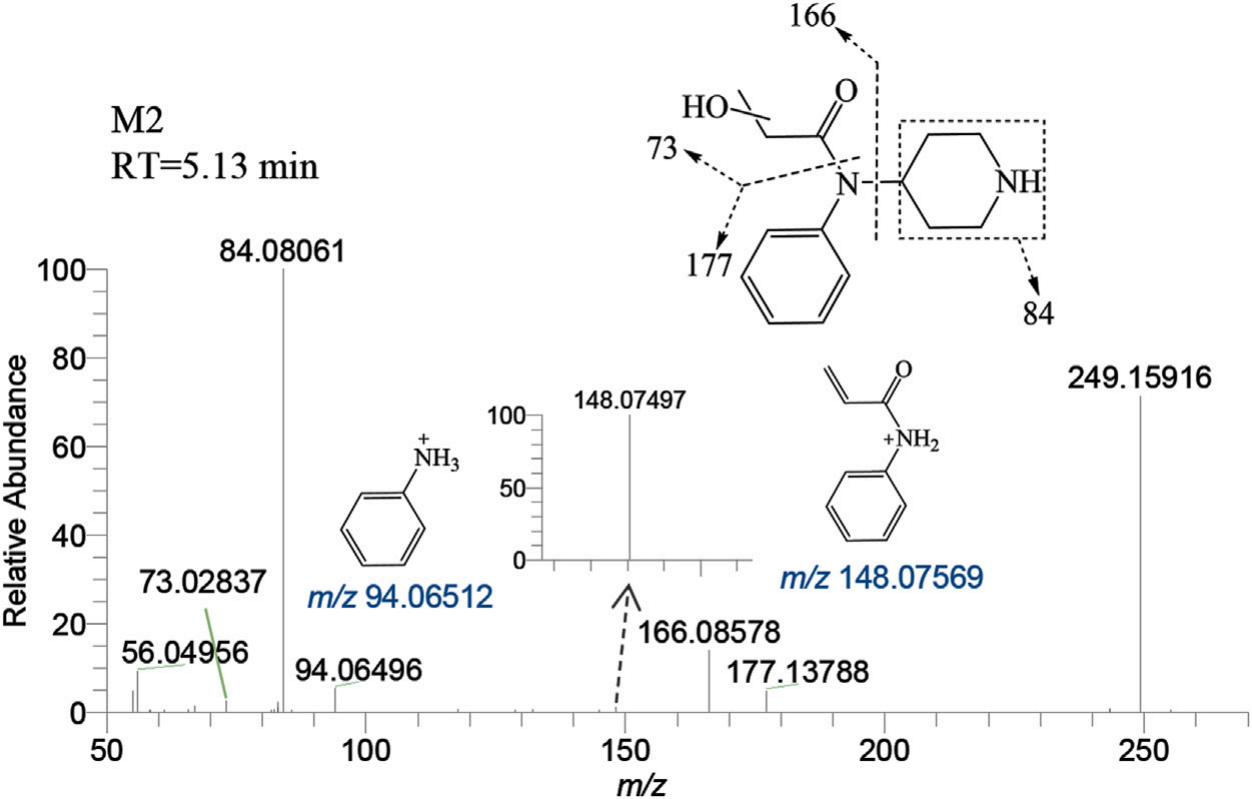
Mass spectra and assigned fragmentation patterns for M2.

M2 was generated by hydroxylation of M1. M3-a, M3-b, M3-c, and M3-d were the monohydroxylation metabolites of the parent drug, while dihydroxylation results in M′3-e. M3-a was solely identified in the liver of zebrafish and not in the brain. Conversely, M′3-e was exclusively detected in the brain. M2 was observed at a retention time of 5.13 min, with an accurate mass of the protonated molecular ion [M + H]^+^ at *m/z* 249.15975, corresponding to the molecular formula C_14_H_20_N_2_O_2_. Compared to the characteristic fragments of M1, it was inferred from *m/z* 166.08578, 148.07497 (166→148, a loss of H_2_O) and 73.02837 that the hydroxylation site of M2 was located on the propionyl moiety as shown in Figure 5.

**FIGURE 6:**
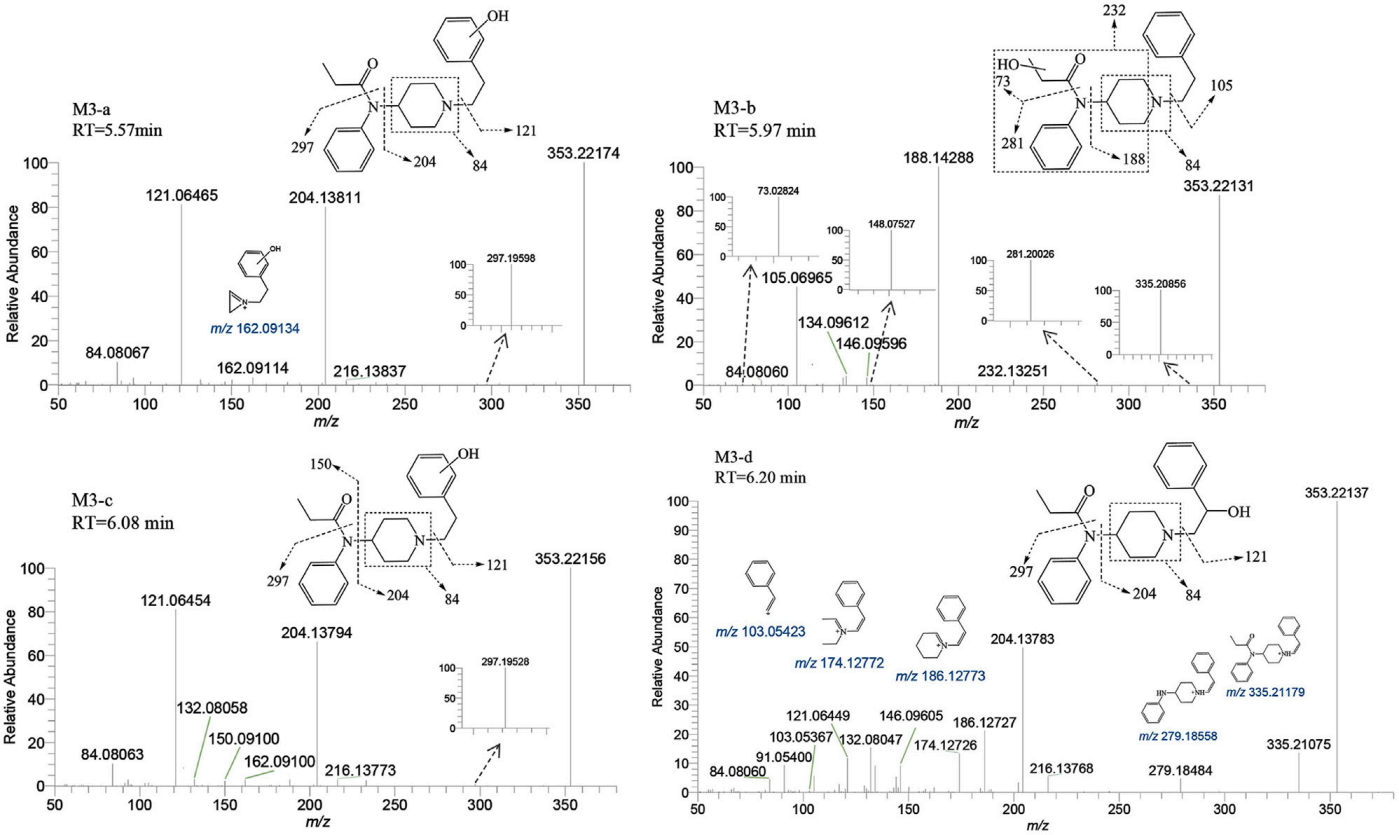
Mass spectra and assigned fragmentation patterns for monohydroxylated metabolites (M3 group).

M3-a, M3-b, M3-c, and M3-d were identified at retention times of 5.57, 5.97, 6.08, and 6.20 min, respectively. These metabolites exhibited an accurate mass of the protonated molecular ion [M + H]^+^ at *m/z* 353.22235 (C_22_H_28_N_2_O_2_). The MSMS spectrum of M3 group is depicted in Figure 6. The presence of characteristic fragment ion peaks at *m/z* 84, 121, 162, and 204, along with the absence of *m/z* 335 (a loss of H_2_O), in both M3-a and M3-c, suggest that a monohydroxylation reaction occurred on the benzene ring of phenylethyl moiety in each metabolite.

The variation in retention times across these metabolites indicates distinct reaction sites for each hydroxylation event. The fragment ions of M3-b at *m/z* 73.02824, 148.07527, 232.13251, and 335.20856 indicate that the monohydroxylation reaction occurred on the propionyl moiety. For metabolite M3-d, the ion fragments adhered to a fragmentation pattern of *m/z* 353→204→186 and 353→335→279→186→174 (Cooman et al., 2022a). The transitions of *m/z* 353→335 and 204→186 were indicative of dehydration, thereby suggesting that the hydroxylation reaction site for M3-d is situated on the alkyl portion of the phenethyl moiety. Through detailed analysis of the tandem mass spectrometry data, it has become apparent that the MSMS spectrum alone is insufficient to pinpoint the exact carbon atom undergoing hydroxylation. However, considering the inherent instability of α-carbon hydroxylation in fentanyl metabolites, we infer that the hydroxylation occurs at the more stable β-carbon position. This deduction is supported by both the spectral data and the chemical logic pertaining to the likely metabolic pathways.

**FIGURE 7:**
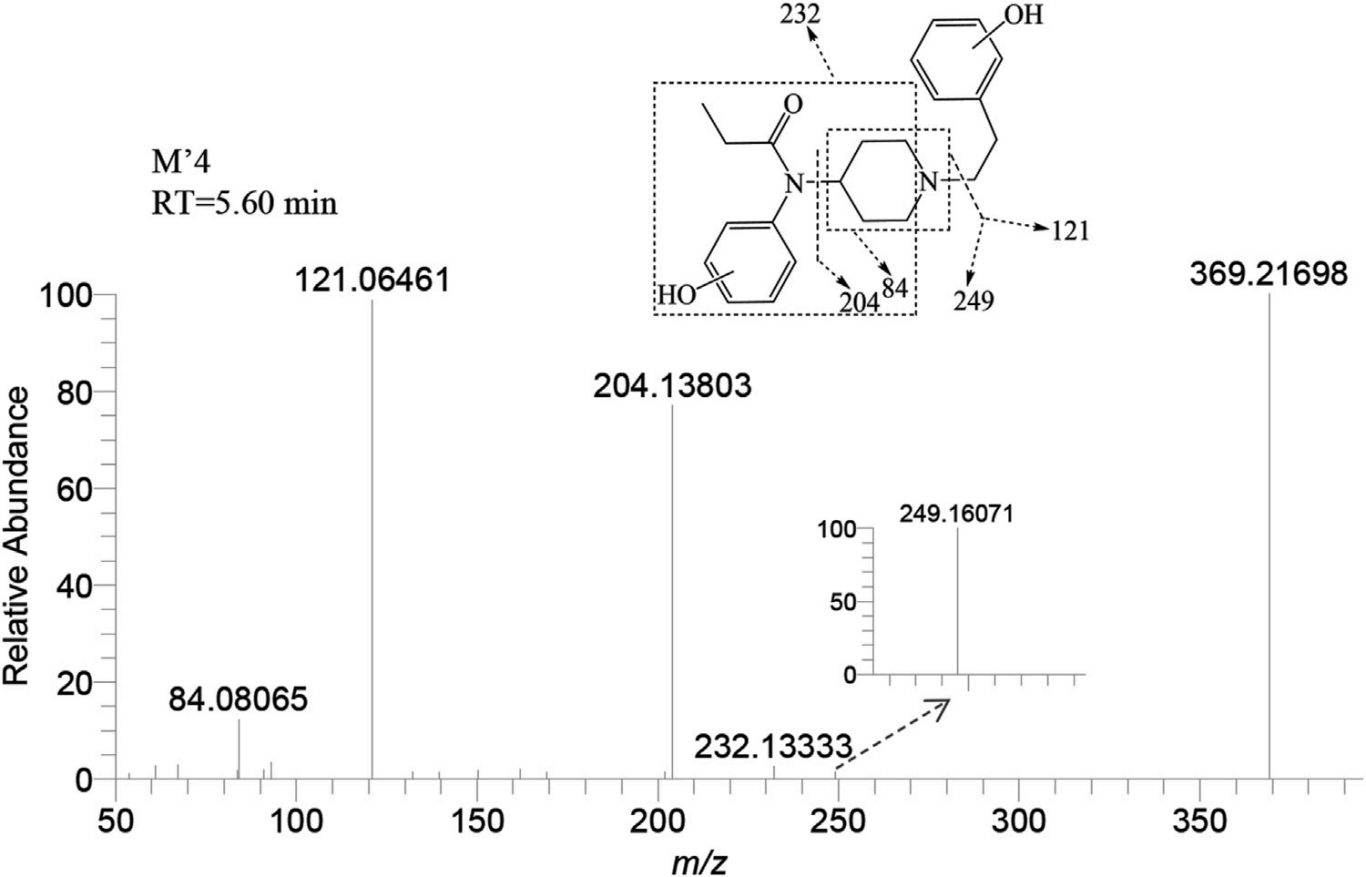
Mass spectra and assigned fragmentation patterns for M′4.

M’4 was exclusively observed in the brain of zebrafish at a retention time of 5.60 min. It formed an accurate mass of the protonated molecular ion [M + H]^+^ at *m/z* 369.21727 (C_22_H_28_N_2_O_3_). The absence of product ions at *m/z* 351 and 333, due to the loss of H_2_O, indicates that M′4 underwent hydroxylation on the benzene ring of fentanyl. The presence of the characteristic fragment ions at *m/z* 121.06461, 204.13803, and 84.08065 strongly suggest that one hydroxylation site is on the benzene ring of phenylethyl moiety. This observation exclude the possibility of a second hydroxy group being present on the phenylethyl moiety, as shown in Figure 7. Similarly, it can be inferred that another hydroxylation site could be located on the benzene ring of the aniline group from the fragment ions *m/z* 232.13333 and 249.16071.

#### 3.2.2 Examination of phase II metabolites

Ten phase II metabolites were identified in the liver of zebrafish, contrastingly, only two (M5 and M9-a) were detected in the brain.

##### 3.2.2.1 Metabolites of methylation

**FIGURE 8:**
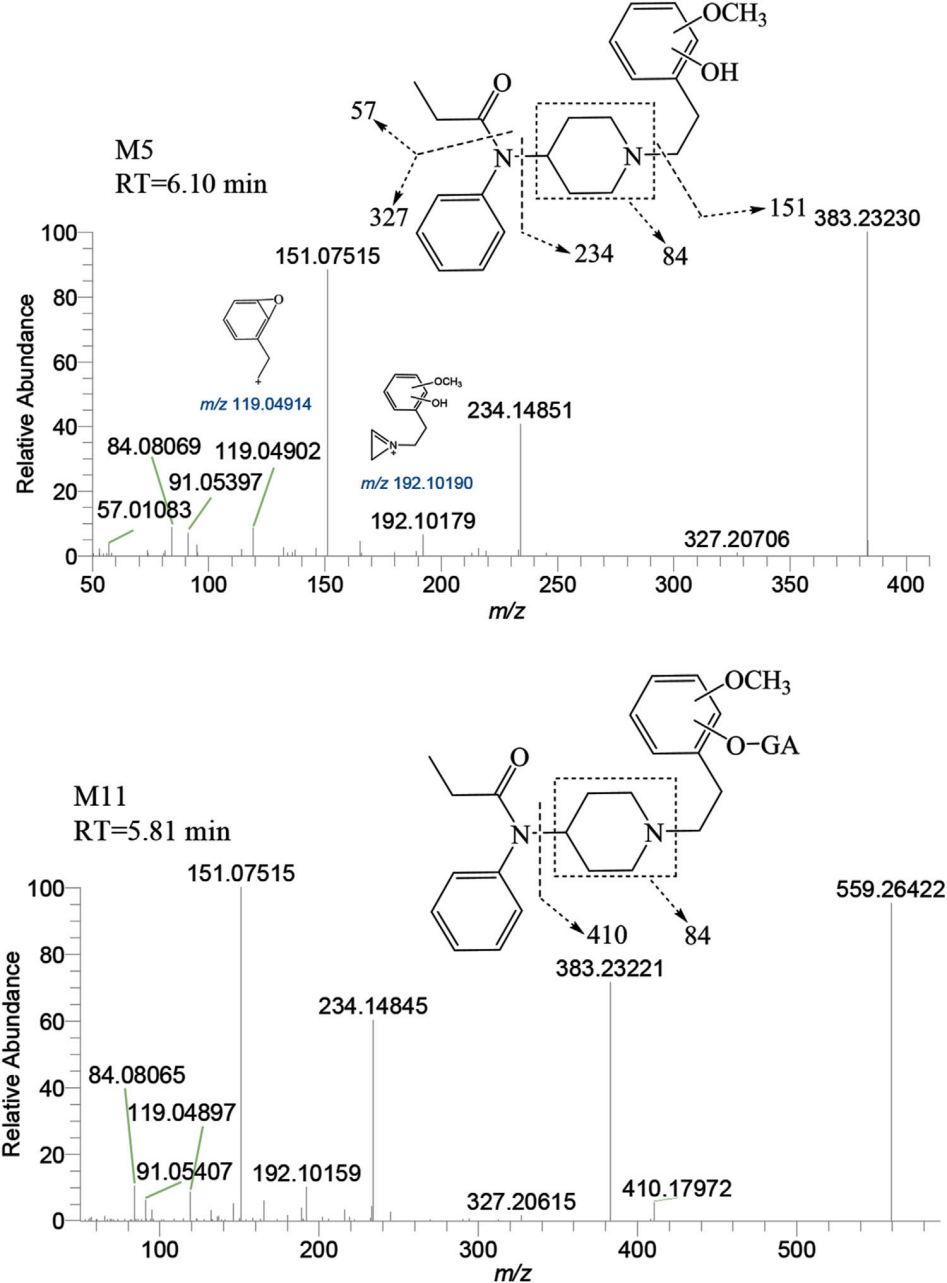
Mass spectra and assigned fragmentation patterns for methylated metabolites (M5 and M11).

M5 was observed at a retention time of 6.10 min, with an accurate mass of the protonated molecular ion [M + H]^+^ at *m/z* 383.23292 (C_23_H_30_N_2_O_3_). Similar to M′4, the absence of product ions at *m/z* 365, 351, and 333, which would indicate the loss of H_2_O, was also noted for M5. The presence of characteristic fragment ions at *m/z* 151.07515, 234.14851, 119.04902, 192.10179, and 57.01083 suggests that the dihydroxylation reaction occurred on the benzene ring of the phenylethyl moiety. The specific fragment at *m/z* 192 supports the inference that a catechol metabolite was formed on the benzene ring of the phenylethyl moiety. Subsequently, a hydroxyl moiety underwent a methylation reaction formed M5. M11 was characterized by a retention time of 5.81 min with an accurate mass of the protonated molecular ion [M + H]^+^ at *m/z* 559.26501 (C_29_H_38_N_2_O_9_). From the characteristic fragment ions *m/z* 151.07515, 383.23221, 234.14845, and 192.10159, it can be inferred that M11 was formed by the glucuronidation of another hydroxyl moiety on M5. The MSMS spectrum of methylated metabolites are displayed in Figure 8.

##### 3.2.2.2 Metabolites of glucuronidation

**FIGURE 9:**
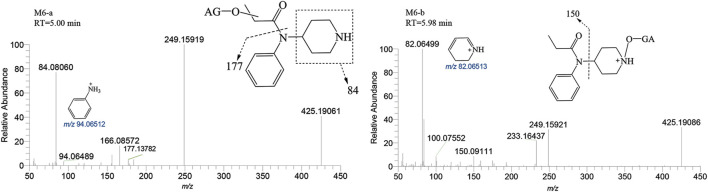
Mass spectra and assigned fragmentation patterns for M6 group.

**FIGURE 10:**
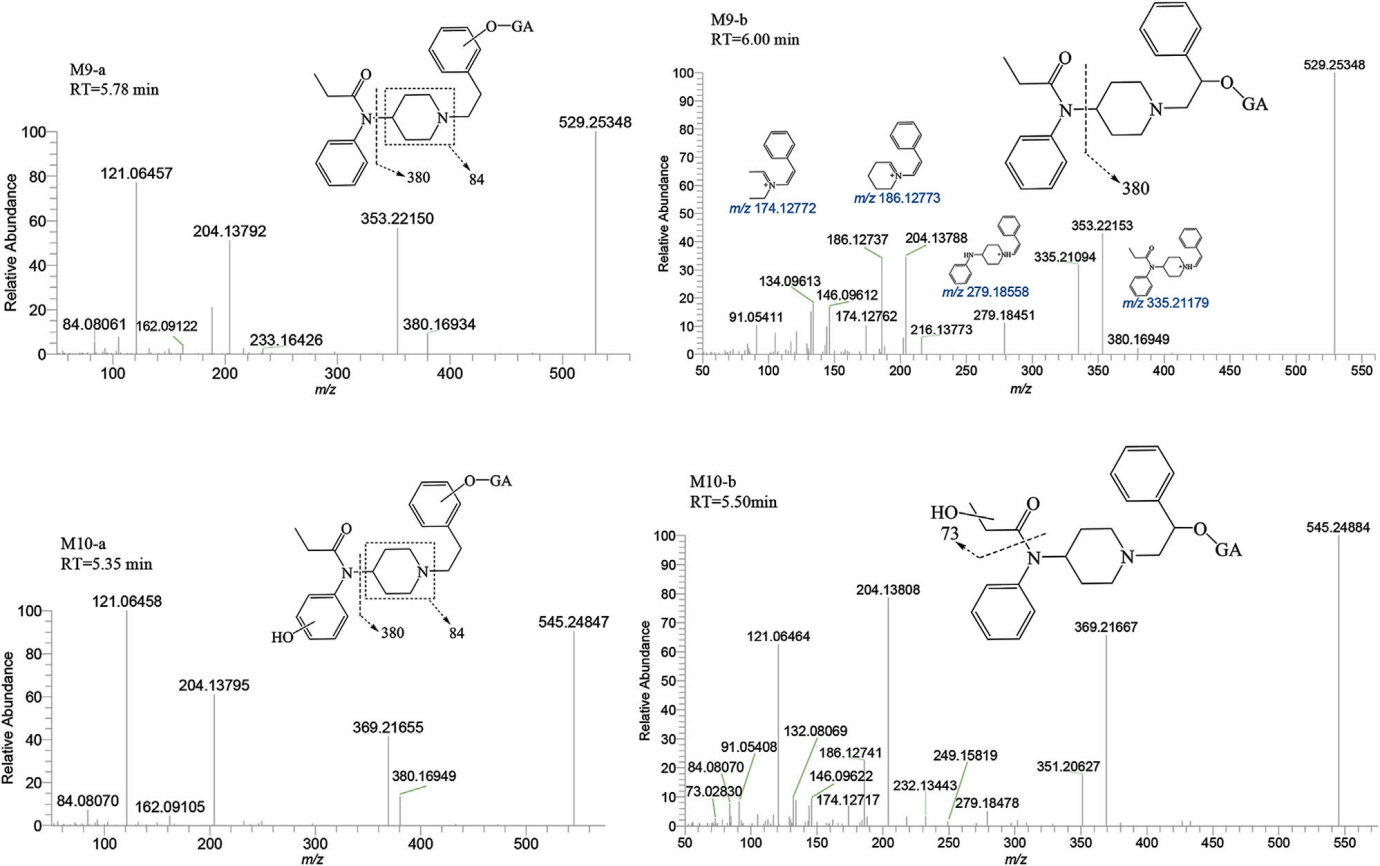
Mass spectra and assigned fragmentation patterns for glucuronidated metabolites (M9 and M10 group).

M6-a and M6-b were glucuronidated metabolites, formed through dealkylation and hydroxylation of the piperidine ring. They were identified with retention times of 5.00 and 5.98 min, respectively. Their protonated molecular ion [M + H]^+^ was observed at an accurate mass of *m/z* 425.19184 (C_20_H_28_N_2_O_8_) as shown in Figure 9. The formation of M6-a from M2 through glucuronidation can be inferred from the characteristic fragment ions. The presence of fragment ions *m/z* 82.06499, 249.15921, 233.16437, 150.09111, and 100.07552 suggest that M6-b was formed via glucuronidation after the N atom of the piperidine ring underwent dealkylation and oxidation. M9 and M10 moiety were formed through glucuronidation following the hydroxylation of the parent drug (Figure 10). M9-a and M9-b were identified at the following retention times: 5.78 and 6.00 min, with an accurate mass of the protonated molecular ion [M + H]^+^ at m/z 529.25444 (C_26_H_36_N_2_O_8_). They were formed as a result of glucuronidation, a process that occurred after parent drug underwent monohydroxylation. The MSMS spectrum of M9-a revealed fragment ions at *m/z* 121.06458, 353.22153, 204.13795, 84.08066, 380.16959 and no *m/z* 335 (a loss of H_2_O), suggesting that M9-a resulted from the glucuronidation of either M3-a or M3-c. In contrast, the fragment ions of M9-b at *m/z* 353.22153, 204.13788, 186.12737, 335.22153, and 134.09613 infer that M9-b is a product of the glucuronidation of M3-d. Similarly, M10-a and M10-b were formed through glucuronidation following the dihydroxylation of the parent drug.

##### 3.2.2.3 Metabolites of sulfation

**FIGURE 11:**
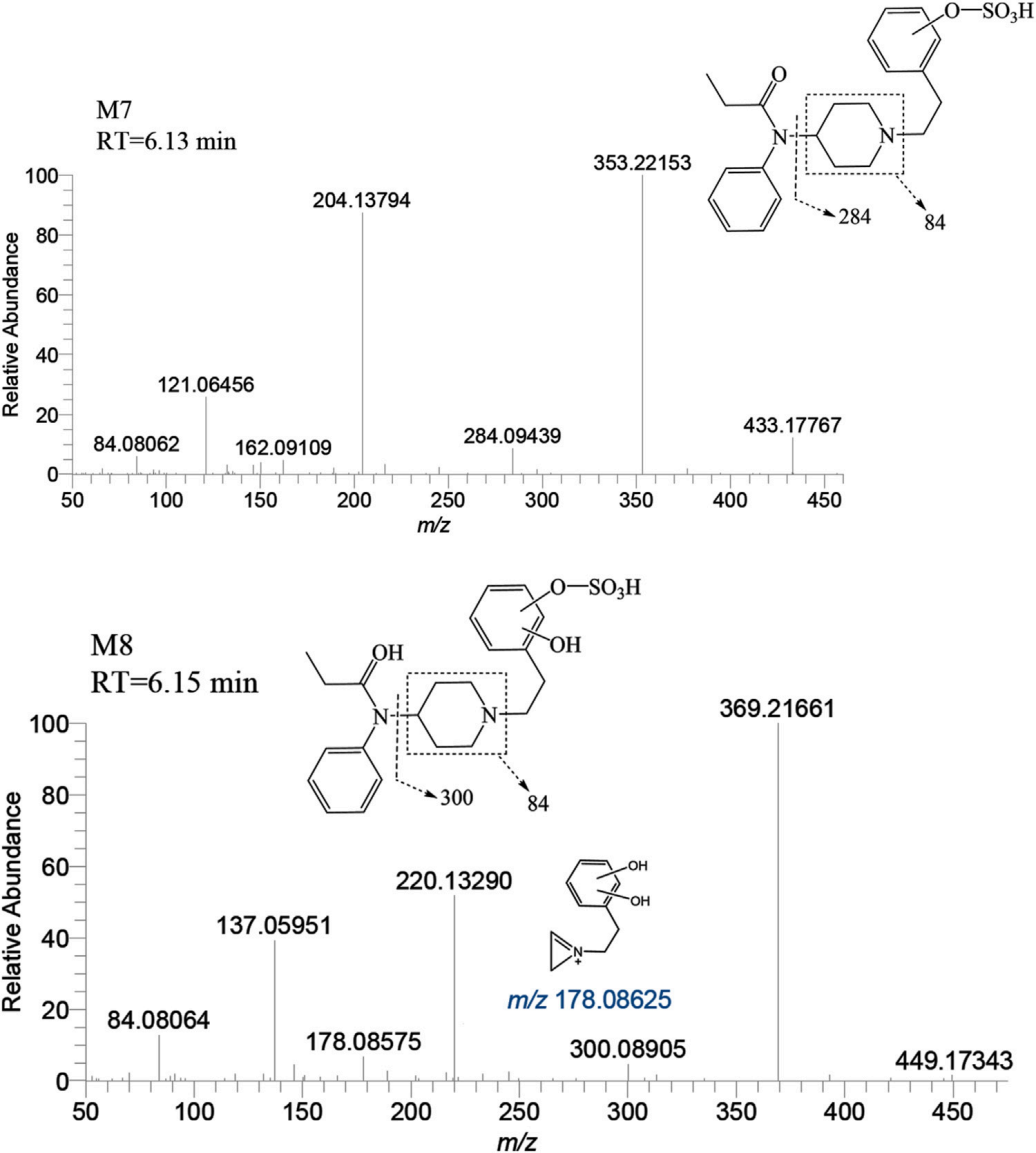
Mass spectra and assigned fragmentation patterns for sulfated metabolites (M7 and M8).

The metabolite M7 was observed with a retention time of 6.13 min, and it formed an accurate mass of the protonated molecular ion [M + H]^+^ at *m/z* 433.17917 (C_22_H_28_N_2_O_5_S). The presence of the characteristic fragment ions at *m/z* 353.22153, 204.13794, 121.06456, 284.09439, 162.09109 and no *m/z* 335 (a loss of H_2_O) was observed indicated that M7 was formed through sulfation, following monohydroxylation of the parent drug, a process likely carried out by M3-a or M3-c. The post-dihydroxylation sulfation product M8, presented a retention time of 6.15 min. Its protonated molecular ion [M + H]^+^ ion was at *m/z* 449.17408, corresponding to the molecular formula C_22_H_28_N_2_O_6_S. The MSMS spectrum of M8 displayed characteristic fragment ions at *m/z* 369.21661, 220.13290, 137.05951, 178.08575 and no *m/z* 431, 351, and 333 by the loss of H_2_O were observed indicating the presence of two hydroxy moiety at the benzene ring of phenylethyl moiety, with one of them underwent sulfation (Figure 11).

The parent drug was found in high abundance in all samples. Upon comparing the peak areas of 17 metabolites in the experiment, ranking peak areas as outlined in Table 1, the most dominant metabolite was the metabolite of monohydroxylation and glucuronidation (M9-a), followed by monohydroxylated metabolite (M3-c, precursor of M9-a) in the zebrafish’s liver, and the highest abundance of normetabolite (M1) was found in the zebrafish’s brain, followed by monohydroxylated metabolite (M3-b). The N-dealkylation metabolite on the piperidine ring, also referred to as norfentanyl, has been identified as a signature metabolite of fentanyl. M9-a, M3-b, and M3-c contains all the structural characteristics of fentanyl. In summary, we have discerned that the metabolites, specifically M9-a in conjunction with M3-c, have been identified as potential indicators of fentanyl toxicity within the hepatic system. Simultaneously, we advocate for the combined use of M1 and M3-b metabolites as the metabolic signposts for fentanyl within the cerebrum.

### 3.3 Biotransformation pathways of fentanyl

**FIGURE 12:**
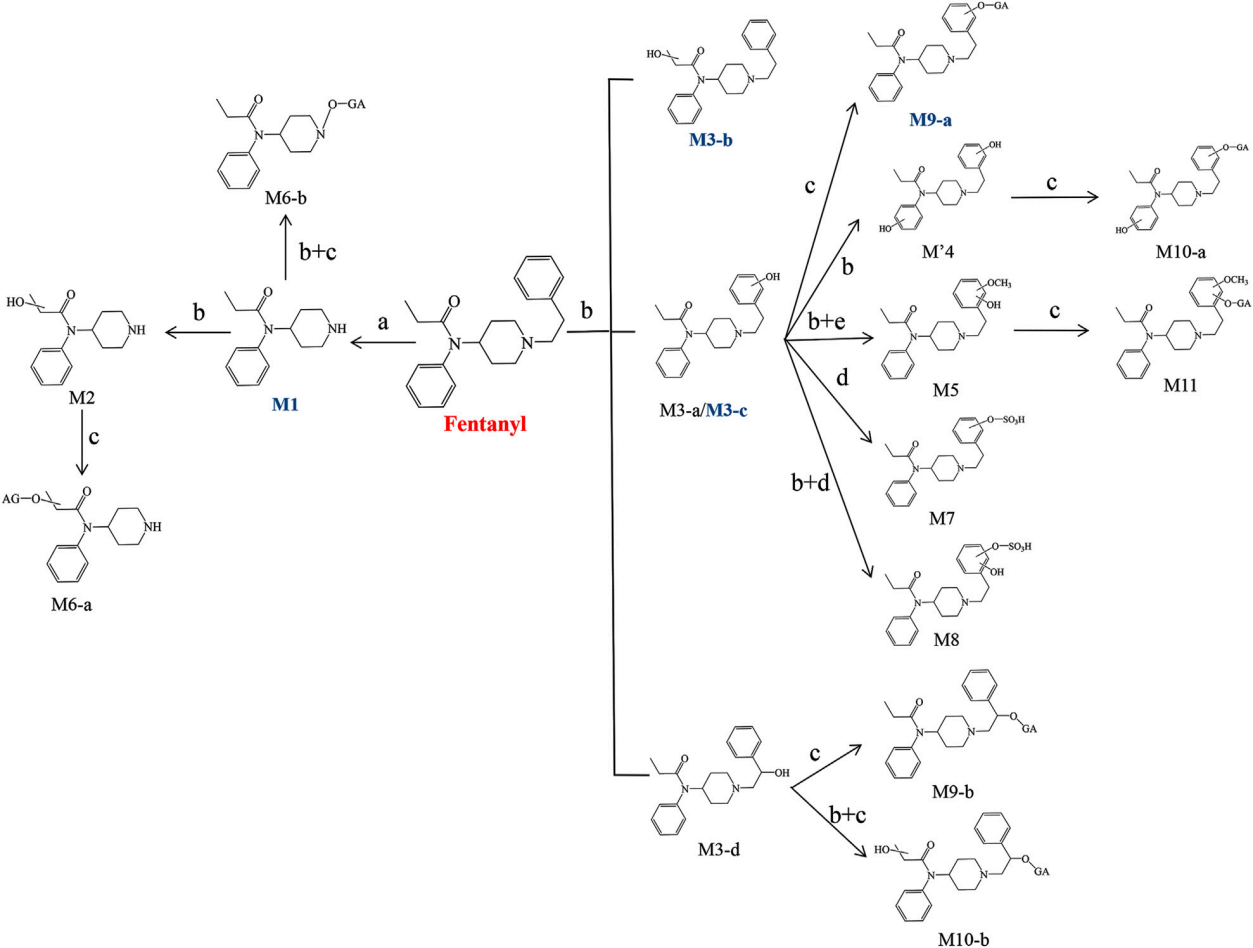
Proposed biotransformation pathways of fentanyl in the liver and brain of zebrafish (a. N-Dealkylation, b. Hydroxylation, c. Glucuronidation, d. Sulfation, e. Methylation). Major metabolites are marked by blue bold.

The structural analysis of various metabolites in the liver and brain of zebrafish has revealed the biotransformation pathways of fentanyl. Metabolites were formed through several processes: N-dealkylation (M1) followed by hydroxylation (M2) and glucuronidation (M6-a); N-dealkylation followed by oxidation at the N atom of the piperidine ring and glucuronidation (M6-b); monohydroxylation (M3-a, M3-b, M3-c, M3-d) followed by glucuronidation (M9-a, M9-b) or sulfation (M7); dihydroxylation (M′4) followed by glucuronidation (M10-a, M10-b) or sulfation (M8); and dihydroxylation at the benzene ring of phenylethyl moiety followed by methylation of one hydroxyl moiety (M5) and glucuronidation of another hydroxyl moiety (M11). Figure 12 shows the proposed biotransformation pathway of fentanyl in the zebrafish’s liver and brain.

## 4 Conclusion

Given the increasing influence of fentanyl on public health, enhancing research into fentanyl metabolism is crucial. In this study, we used a zebrafish animal model, a method that is efficient in terms of time, cost, and sensitivity, in conjunction with UHPLC-QE HF MS, to perform a comparative analysis of the metabolites and biotransformation pathways of fentanyl in the zebrafish’s liver and brain. Our research identified 17 unique metabolites of fentanyl in the liver and brain of the zebrafish, including 7 phase I metabolites and 10 phase II metabolites. Fewer metabolites were detected in the brain than in the liver, the primary metabolic organ. M′4 was found exclusively in the brain, not in the liver. M9-a (monohydroxylation followed by glucuronidation) and M3-c (monohydroxylation) have been discerned as potential harbingers of fentanyl-induced hepatotoxicity. Concurrently, M1 (normetabolite) and M3-b (monohydroxylation) have been identified as cerebral metabolic markers for fentanyl. Notably, the diversity of metabolites identified in this study exceeded prior reports, particularly regarding phase II metabolites, and the sulfation metabolite was identified for the first time. This study provides an in-depth analysis of fentanyl metabolism. These insights could provide valuable evidence for the examination and identification of biological samples in instances of fentanyl misuse and related fatalities.

## Data Availability

The original contributions presented in the study are included in the article/Supplementary Materials, further inquiries can be directed to the corresponding author.
